# Long-term outcome in people who use drugs successfully treated for hepatitis C infection with glecaprevir/pibrentasvir

**DOI:** 10.1016/j.jve.2024.100569

**Published:** 2024-12-03

**Authors:** Shana Yi, David Truong, Brian Conway

**Affiliations:** a Vancouver Infectious Diseases Center, Vancouver, British Columbia, Canada; b Health Sciences, Simon Fraser University, Burnaby, British Columbia, Canada

## Abstract

**Background:**

Several clinical trials, including the recently published the GRAND PLAN study from Vancouver Infectious Diseases Center (VIDC), have demonstrated the efficacy of hepatitis C (HCV) therapy among active drug users, including those facing significant addiction-related and social challenges. In the GRAND PLAN, we documented sustained virological response post-treatment Week12 (SVR12) in 108/117 (92.3 %) individuals (108/111 (mITT) or 97.3 % of those reaching the SVR12 timepoint) receiving an 8-week course of glecaprevir/pibrentasvir (G/P), with almost all using fentanyl and over half being unstably housed. Data on the maintenance of this favorable outcome in the long-term in such a population with a significant risk of reinfection is limited. We hypothesized that the offer of ongoing multidisciplinary care (including addiction care) after SVR12 was achieved would reduce the likelihood of loss to follow up, HCV reinfection or death and consolidate the gains achieved by initial engagement in care to diagnose and treat HCV infection.

**Methods:**

The inception cohort for this analysis was the 108 individuals achieving a cure of HCV infection within the GRAND PLAN study. All were offered the opportunity to continue to receive care at the VIDC. This is a multidisciplinary model of care to address medical, mental health, social and addiction-related concerns on an ongoing basis. This included, if necessary, opiate agonist and safer supply therapy, usually provided by the pharmacy adjacent to our inner-city campus. Among those choosing to be retained in care, the endpoint of this analysis was loss to follow up, mortality and HCV reinfection and their correlates. Reinfection was ascertained by repeat HCV RNA testing every 6 months, more frequently if clinically indicated.

**Results:**

Of the 108 individuals making up the inception cohort for this analysis, all chose to remain in care at the VIDC. We note a median age of 47 (22–75) years, 28 % female, 21.3 % identifying as indigenous, the majority with mild fibrosis (90.8 % F0–F2), slightly more than half with unstable housing. It is of note that we recorded a 20 % decrease in fentanyl users among those who were cured compared to the baseline evaluation of the overall study cohort (73.5 % vs 94.9 %, p < 0.000001). Among the cured individuals, 104 (96.3 %) remained alive, while 4 individuals died of opioid overdoses. Out of the 104 , 99 (95.2 %) remained HCV-free, while 5 (4.8 %) were re-infected. All five have recently initiated repeat HCV therapy at VIDC, 2 of whom are already documented to be cured.

**Conclusion:**

Among a population of vulnerable inner-city residents cured of HCV infection within a multidisciplinary program of care at the VIDC, all individuals accepted the offer to remain in long-term follow up, with a statistically significant reduction in fentanyl use over time. In the setting of an ongoing opioid crisis where 3 deaths/day are recorded in the neighborhood where the study population resides, we documented 4 deaths. Reinfections occurred at a very modest rate, with maintenance in care allowing prompt re-treatment, with a cure already being documented in 2/5 cases, with the other 3 individuals remaining on HCV therapy at the VIDC.

## 1 Introduction

Hepatitis C Virus (HCV) infection represents a major global health challenge, particularly among populations engaged in high-risk behaviors such as active drug use. Globally, HCV infection remains a significant public health issue, with an estimated 71 million people living with chronic HCV infection.^1^ Injection drug use is one of the major drivers of the epidemic, accounting for a substantial proportion of new infections. It is estimated that between 60 % and 80 % of people who inject drugs (PWID) are infected with HCV, reflecting both the high risk of transmission through shared needles and the chronic nature of the disease.^2^ The World Health Organization (WHO) has identified PWID as a key population for targeted HCV interventions, given the high prevalence and incidence rates within this group.^3^ Historically, the management and treatment of HCV in active drug users have faced numerous obstacles, including poor adherence to treatment regimens, high rates of reinfection after a cure have been achieved, and significant addiction-related and social challenges. It is estimated that over 15.6 million people inject drugs in the world, with 6.1 million living with HCV infection.^1^ Recent advances in antiviral therapies have shown promising results, offering hope for effective management and eradication of HCV within this population.

In Canada, the burden of HCV among PWID mirrors global trends. Approximately 250,000 Canadians are living with chronic HCV, with PWID representing a significant proportion of this population.^4^ The prevalence of HCV among PWID in Canada is estimated to be about 66 %, highlighting the urgent need for effective prevention, treatment, and harm reduction strategies to control transmission. National initiatives such as the Canadian Network on Hepatitis C (CanHepC) have been instrumental in advancing research and policy efforts to address HCV among high-risk groups, including PWID. However, challenges persist, including regional disparities in access to treatment and access to harm reduction services, as well as ongoing social and structural determinants that contribute to HCV prevalence among drug users. Vancouver, particularly the Downtown Eastside (DTES) neighborhood, has been recognized as an epicenter of the HCV epidemic in Canada. The prevalence of HCV in this area is alarmingly high, with estimates suggesting that up to 70 % of PWID in the DTES are HCV positive. This high prevalence is compounded by significant rates of homelessness, mental health issues, and polysubstance use, including the widespread use of fentanyl.

Several studies have highlighted the efficacy of direct-acting antivirals (DAAs) in treating HCV among active drug users. DAAs have revolutionized HCV treatment due to their high cure rates, shorter treatment durations, and improved safety profiles compared to previous interferon-based therapies.^5^, ^6^, ^7^, ^8^, ^9^, ^10^ The combination of glecaprevir/pibrentasvir (G/P) has emerged as a particularly effective regimen. G/P is administered as three tablets once daily with food with pan-genotypic activity against HCV. This regimen is administered for 8 weeks to treatment-naive non-cirrhotic and compensated cirrhotic individuals with success rates exceeding 95 % among those who complete treatment across multiple clinical trials.^6^^,^^8^^,^^9^^,^^11^, ^12^, ^13^

The GRAND PLAN study, recently published by the VIDC, adds to the growing body of evidence supporting the use of G/P in active drug users.^14^ This study documented an SVR12 rate of 92.3 % among individuals, with a modified intention-to-treat (mITT) analysis showing a 97.3 % (108/111 individuals) success rate among those who reached the sustained virological post-treatment Week 12 (SVR12) timepoint. Notably, the majority of individuals in the GRAND PLAN study were active fentanyl users, and over half were unstably housed, underscoring the potential of G/P to achieve high cure rates even in the most marginalized populations.

Despite these encouraging results, data on the long-term maintenance of such positive outcomes remains limited. Active drug users are at a significant risk of HCV reinfection due to ongoing high-risk behaviors. Therefore, it is crucial to develop strategies that not only achieve SVR but also sustain these outcomes over time. The integration of ongoing multidisciplinary care, including addiction services, post-SVR, is hypothesized to be a critical factor in reducing loss to follow-up, preventing reinfection, and lowering mortality rates. By consolidating the gains achieved through initial HCV treatment and addressing the broader health and social needs of this population, sustained multidisciplinary engagement holds promise for improving long-term outcomes and reducing the burden of HCV among active drug users. This analysis was conducted to evaluate the long-term outcome of individuals having achieved a cure of HCV infection within the GRAND PLAN study, with an emphasis on the maintenance of cure and mortality as primary outcomes.

## 2 Methods

### 2.1 Study design and individuals

The GRAND PLAN study results stand as published.^14^ Briefly, non-cirrhotic HCV-infected treatment-naïve adults were offered therapy with glecaprevir/pibrentasvir (G/P) given as 3 tablets once daily with food. Treatment was administered within the context of a multidisciplinary program of care, to address all medical, social, mental health and addiction-related needs on a longitudinal basis. HCV therapy was administered with the support of a central pharmacy, with weekly/daily dispensing available as required. Key baseline characteristics (n = 117) included: median age of 46 (range 22–75) years old, with 27 % of subjects identified as female and 21.4 % identified as indigenous. The majority of individuals were carrying either genotype 1 (56.4 %) or 3 (32.5 %). Almost half (48.7 %) were not in stable housing, living in shelters that needed to be vacated during the day or short-term rooms needing to be vacated in the coming weeks or few months. Active drug use (defined as using or injecting >2 days in the previous week) was confirmed in over 95 % of cases. At the SVR12 time point, 108 out of 111 subjects (97.3 %) were cured.

We followed upindividuals who had achieved SVR12 (2019–2022). All 108 individuals were offered the opportunity to engage in long-term follow-up at the VIDC, where they would continue to receive multidisciplinary care. HCV RNA testing was performed every 6 months to document preserved cure or recurrent viremia. Testing could be performed more frequently in the setting of suspected acute hepatitis. If viremia was detected, a new course of HCV therapy was offered at the VIDC. The primary outcome of this analysis was detection of viremia and its correlates, as well as mortality.

All participants provided informed consent before any study procedures were performed. The study protocol was approved by Advarra and done according to the Declaration of Helsinki and International Conference on Harmonization good clinical practice guidelines.

### 2.2 Statistical analysis

In this analysis, descriptive statistics was used to report on the primary outcome and all secondary outcomes. There was no randomization, blinding or control groups in this study. As appropriate, correlates of unfavorable outcomes were evaluated. Univariable comparisons (e.g. t-tests) were calculated utilizing the GraphPad Prism 5.1 Software (GraphPad Software, Inc., La Jolla, CA, USA).

## 3 Results

Our inception cohort for this analysis consisted of 108 subjects who had achieved SVR from the GRAND PLAN study. The original study demographics are shown in Table 1, the demographics of the follow-up cohort in Table 2.We note: a median age of 47 (22–75) years old, with 28 % of subjects identified as female and 21.3 % identified as indigenous and 75 % identified as Caucasian. The majority of individuals had mild fibrosis (90.8 % F0–F2 by FibroScan measure). Slightly more than half had unstable housing, a higher proportion than in the original cohort, while 96.3 % were active drug users. It is important to note that from this follow-up analysis cohort, there was a 20 % decrease in fentanyl users among those who were cured compared to baseline evaluation of the overall cohort study resulting in a (73.5 % vs. 94.9 %, P < 0.000001).

**Table 1:** Characteristics of the total glecapravir/pibrentasvir cohort.

Demographics	N = 117
**Age (Range)**	46 (22–75)
**Sex**	
Female	32 (27.4 %)
Male	85 (72.6 %)
**Ethnicity**	
Caucasian	87 (74.4 %)
Indigenous	25 (21.4 %)
Other	3 (2.6 %)
**Genotype**	
1	66 (56.4 %)
2	13 (11.1 %)
3	38 (32.5 %)
**Fibrosis Stage**	
F0-F2	107 (91.5 %)
F3-F4	10 (8.5 %)
**Alcohol Use**	
Yes	36 (30.8 %)
No	81 (69.2 %)
**Smoking Status**	
Yes	99 (84.6 %)
No	18 (15.4 %)
**Active Drug User**	112 (95.7 %)
**Drug Use Profile**	
Amphetamines	57 (48.7 %)
Cocaine	31 (26.5 %)
Fentanyl	111 (94.9 %)
Methadone	49 (41.9 %)
Benzodiazepine	15 (12.8 %)
Morphine	41 (35 %)
**Unstable Housing**	57 (48.7 %)
**HIVco-infected**	3 (2.6 %)

Table 1. Baseline characteristics of individuals who filled out the questionnaire during CPC. Unstable housing is defined by living in a single room occupancies (SRO's), shelters, or homelessness.

**Table 2:** Characteristics of thefollow-up cohort (GRAND PLAN study).

Demographics	N = 108
**Median Age (Range)**	47 (22–75)
**Sex**	
Female	30 (28 %)
male	78 (72.2 %)
**Ethnicity**	
Caucasian	81 (75 %)
Indigenous	23 (21.3 %)
Other	4 (3.7 %)
**Fibroscan Score**	
F0-F2	98 (90.8 %)
F3-F4	10 (9.2 %)
**Unstable Housing**	57 (52.8 %)
**Substance Use**	
Fentanyl	79 (73.5 %)
cocaine	28 (26.5 %)
Amphetamines	53 (48.7 %)
**Active Drug User**	104 (96.3 %)

Table 2. Baseline characteristics of follow-up participants who filled out the questionnaire during CPC. Unstable housing is defined by living in a single room occupancies (SRO's), shelters, or homelessness.

**Fig. 1:**
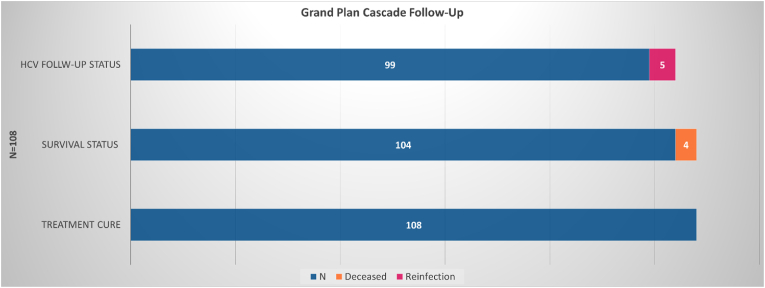
Cascade of care. Fig. 1 Cascade of HCV treatment care of the study cohort showing the progression of mortality and reinfection cases among those who achieved SVR. HCV: hepatitis C; SVR: sustained virological response.

All 108 eligible individuals agreed to remain in long-term follow-up at the VIDC. Of these, 104 (96.3 %) remained alive during the follow=up period, with 4 deaths due to opioid overdoses, occurring at 1–7 months after documentation of HCV cure. Of the remaining 104 subjects, 99 (95.2 %) remained HCV-free while 5 (4.8 %) were re-infected (Fig. 1).

Virologic reinfection was documented in 5 cases summarized in Table 3. These 5 individuals were age 31–67 years, all males with unstable housing, all active opiate/fentanyl users. These cases occurred 5–13 months after a cure had been achieved. In the overall cohort, a total of 216 person-years of follow-up has been documented. By these parameters, the overall rate of reinfection was 2.3/100 person years.

**Tabl:** Subjects with documented hepatitis C reinfection.

Subject	Sex	Age	Ethnicity	Fibroscan Score	Drug User	Type of Drug	Unstable Housing	HCV Cure SVR 12 date	Reinfection confirmation Date	Reinfected confirmation RNA	Current RNA status
1	M	68	Indigenous	F1	Y	O	Y	10-Oct-19	26-Feb-20	245	2591
2	M	34	Caucasian	F0	Y	A,O	Y	23-Oct-19	17-Sep-20	79090	79090
3	M	34	Caucasian	F0	Y	A,O,M	Y	28-Feb-20	31-May-20	552987	CURED S/V
4	M	43	Indigenous	F0	Y	A,O,M,B	Y	27-Feb-20	10-Dec-20	7688	CURED S/V
5	M	32	Caucasian	F1	Y	O	Y	13-Jan-20	24-May-22	94890	94890

∗O=Opiates, A = Amphetamines, M = Methadone, B=Benzodiazepines, MOR=Morphine.

∗∗ Both subject 3 and 4 have been retreated with sofosbuvir/velpatasvir and are now cured.

Table 3. Baseline characteristics of subjects with documented reinfection and their corresponding viral loads and genotype of reinfection.

For all cases of reinfection, first line HCV treatment is made available through government funding, free of charge, as soon as requested. This was offered in all 5 cases within our program, and all accepted to receive it. To date, 2 individuals have received a 12-week course of sofosbuvir and velpatasvir, with a cure being documented in both cases. The other 3 individuals remain in care and there is a plan to provide them with HCV treatment in the short term.

## 4 Discussion

The World Health Organization's (WHO) global hepatitis strategy, supported by all member states, aims to reduce new hepatitis infections by 90 % and deaths by 65 % between 2016 and 2030.^3^ The availability of highly potent, safe, and easily administered oral agents for HCV treatment is essential but not sufficient to achieve these goals. There is a need to develop systems of care to identify infected individuals, particularly those who do not traditionally engage in healthcare. There is a further need to develop strategies to provide HCV therapy and maximize the likelihood of adherence to therapy and achievement of cure. If this outcome is reached, it may also be important to implement programs to maintain cure and achieve other benefits of continued engagement in care, particularly in vulnerable populations.

To date, many trials and real-world datasets have shown that HCV cure can be achieved in active drug users at rates that are equivalent to those reported in phase 3 clinical trials. The seminal SIMPLIFY study showed SVR rates of 94 % (107/113 individuals) with the use of sofosbuvir and velpatasvir among active drug users, with no cases of virologic failure. Further supporting these findings, this study from Austria conducted by Schmidbauer et al. implemented a directly observed therapy approach, where 145 patients received HCV treatment along with their opioid substitution therapy under medical supervision.^15^ The study found that directly observed therapy was highly effective, with 94.6 % of the high-risk PWIDs achieving SVR. This success rate was comparable to the 97.2 % SVR observed in patients who were presumed to have excellent compliance and self-managed their treatment at home. These results highlight the potential of directly observed therapy in ensuring successful HCV treatment outcomes in challenging patient populations.

In our GRAND PLAN study, we have shown a cure rate of 95 % in a cohort of 118 inner city residents, all of whom were active drug users and half of whom were unstably housed. There are limited data on the long-term outcome of such vulnerable individuals in terms of survival and maintenance of cure.

In this analysis, we show that if individuals are provided HCV therapy in a multidisciplinary program designed to meet their needs, they are favorable to remaining in care once a cure is achieved. In fact, all 108 cured individuals chose to do so. This represents a real strength of our program. A further strength seems to be a reduction in the rate of reinfection compared to that reported in other cohorts. Reinfection following successful HCV therapy among drug users is a well-documented outcome. Meta-analyses have reported reinfection rates of 2–5 cases per 100 person-years, although some real-world datasets indicate rates as high as 20 cases per 100 person-years or more.^16^, ^17^, ^18^, ^19^, ^20^, ^21^, ^22^ We feel that maintenance in care has allowed us to engage in an ongoing dialogue with our participants about safer drug use practices and is the mechanism by which such a low reinfection rate can be achieved. Further evidence of the adoption of safer practices is the statistically significant reduction in fentanyl use. Indeed, we document a substantial decrease in fentanyl use among those who achieved SVR in the GRAND PLAN study, from 94.9 % at baseline to 73.5 % in the follow-up cohort (P < 0.000001). Further, we document a small number of overdose-related deaths we have observed, given the ongoing opioid crisis in our province with over 6 reported deaths/day.

Our study has several significant strengths. A key strength is the long-term follow-up of a very vulnerable patient population having achieved a cure for HCV infection. To our knowledge, this is among the first long-term reports of a cohort of this type and size using a systematic framework such as the one we have implemented. We have also demonstrated additional benefits of our program, such as reduced mortality and safer drug use. Moreover, it is important to highlight that over 20 % of our study population identified as indigenous. In both British Columbia and Canada as a whole, indigenous men and women are disproportionately represented in urban areas and are more likely to be disengaged from healthcare services.^23^^,^^24^ A significant strength of our approach lies in our ability to identify, treat, and cure these individuals. Increasing their participation in our program and ensuring they receive the healthcare services they need and deserve remains a top priority for us.

Our study has several limitations. Notably, cirrhotic individuals were excluded. This exclusion was necessary because, at the time the original GRAND PLAN study was designed, glecaprevir/pibrentasvir (G/P) had not yet been approved for the treatment of cirrhosis. Additionally, the study was conducted at a single site, which may raise concerns about the influence of unique site- or city-specific factors on its success. However, we contend that our programmatic approach is well-documented and could be easily replicated in similar settings.

## 5 Conclusions

Among a population of vulnerable inner-city residents who were cured of HCV infection within a multidisciplinary program at the VIDC, every individual accepted the offer to remain in long-term follow-up care. This commitment resulted in a statistically significant reduction in fentanyl use over time. In the context of an ongoing opioid crisis, only 3 overdose-related deaths were documented. Reinfections occurred at a very modest rate, with maintenance in care also facilitating prompt re-treatment. This study underscores the importance of comprehensive, long-term care programs for maintenance of health among vulnerable populations. As such, this model can serve as a blueprint for similar programs aiming to address HCV infection among vulnerable inner-city populations and achieve additional health benefits in doing so. Additionally, this study serves as a model for maintenance of health among vulnerable populations and improvements of public health. This may contribute to the broader goal of public health improvement, ultimately paving the way for a healthier and more resilient society.

## CRediT authorship contribution statement

**Shana Yi:** Writing – review & editing, Writing – original draft, Visualization, Formal analysis, Data curation, Conceptualization. **David Truong:** Writing – review & editing, Writing – original draft, Supervision, Investigation. **Brian Conway:** Writing – review & editing, Methodology, Investigation, Conceptualization.

## Role of the funding source

The study was funded by a research grant from 10.13039/100019211AbbVie Canada to VIDC. No antiviral medications were provided. The funder had no role in the analysis or interpretation of the study results and did not have access to the raw data. VIDC staff designed and implemented the study and evaluated its outcome under the supervision of Brian Conway.

## Declaration of competing interest

Dr. Conway has received research grants, honoraria and/or acted as a remunerated advisor for 10.13039/100006483AbbVie, Astra Zeneca, Gilead Sciences, GSK, Indivior Canada, 10.13039/100004334Merck, 10.13039/100019533Moderna, 10.13039/100014588Sanofi Pasteur, 10.13039/501100023280Seqirus, and 10.13039/100010877ViiV Healthcare. In particular, 10.13039/100006483AbbVie and Gilead Sciences have funded the community pop-up clinic program in a direct way.

SY has no conflict of interest to declare.

DT has received honoraria and acted as a renumerated advisors for AbbVie and Gilead Sciences.

## Data Availability

Data will be made available on request.
